# Developing a Data Integrated COVID-19 Tracking System for Decision-Making and Public Use

**DOI:** 10.23889/ijpds.v5i4.1389

**Published:** 2020-09-28

**Authors:** A Krusina, O Chen, LO Varela, C Doktorchik, V Avati, S Knudsen, DA Southern, C Eastwood, N Sharma, T Williamson

**Affiliations:** Centre for Health Informatics, Cumming School of Medicine, University of Calgary; W21C Research and Innovation Centre, O’Brien Institute for Public Health, Cumming School of Medicine, University of Calgary

## Abstract

**Introduction:**

The unprecedented COVID-19 pandemic unveiled a strong need for advanced and informative surveillance tools. The Centre for Health Informatics (CHI) at the University of Calgary took action to develop a surveillance dashboard, which would facilitate the education of the public, and answer critical questions posed by local and national government.

**Objectives:**

The objective of this study was to create an interactive method of surveillance, or a “COVID-19 Tracker” for Canadian use. The Tracker offers user-friendly graphics characterizing various aspects of the current pandemic (e.g. case count, testing, hospitalizations, and policy interventions).

**Methods:**

Six publicly available data sources were used, and were selected based on the frequency of updates, accuracy and types of data, and data presentation. The datasets have different levels of granularity for different provinces, which limits the information that we are able to show. Additionally, some datasets have missing entries, for which the “last observation carried forward” method was used. The website was created and hosted online, with a backend server, which is updated on a daily basis. The Tracker development followed an iterative process, as new figures were added to meet the changing needs of policy-makers.

**Results:**

The resulting Tracker is a dashboard that visualizes real-time data, along with policy interventions from various countries, via user-friendly graphs with a hover option that reveals detailed information. The interactive features allow the user to customize the figures by jurisdiction, country/region, and the type of data shown. Data is displayed at the national and provincial level, as well as by health regions.

**Conclusion:**

The COVID-19 Tracker offers real-time, detailed, and interactive visualizations that have the potential to shape crucial decision-making and inform Albertans and Canadians of the current pandemic.

## Introduction

COVID-19 (also known as coronavirus disease) is a respiratory disease caused by the SARS-CoV-2 (Severe Acute Respiratory Syndrome CoronaVirus 2) [1]. The first case was reported in Alberta, Canada on March 5th, 2020; just six days later, the World Health Organization declared the outbreak of COVID-19 a global pandemic [2, 3]. Many countries have had significant outbreaks before the virus reached Alberta. Because of these outbreaks, various countries implemented policies that were put in place to reduce the spread of the virus, such as mandating the closure of all non-essential services, implementing social distancing rules, and affecting city-wide lockdown procedures.

In light of the pandemic, Albertan and Canadian policy-makers reached out to the Centre for Health Informatics (CHI) at the University of Calgary and requested ongoing surveillance information about the spread of COVID-19. At the time, there wasn’t any dashboard that compared the provinces, which was valuable to those making decisions, and the CHI was well-equipped to develop such a tool as well as appropriately positioned to collect and sort the overwhelming amount of information from a variety of sources. As a result, a multidisciplinary team of software developers, epidemiologists, designers, visualization experts, and statisticians was assembled to create a COVID-19 dashboard or “Tracker”. The Tracker was then streamlined to one website for a standardized source of education and information (access at https://www.chi-csm.ca/).

The Tracker visualizes various components of the current COVID-19 pandemic and includes the policy interventions of various countries alongside case count. All graphs and figures on the Tracker are specifically designed with a sense of visual clarity to ensure policy-makers and the general public can easily interpret the data. From this, policy-makers can be well informed to make appropriate decisions that will affect the health and well-being of the broader population, or gauge the efficacy of interventions.

## Methods

### Data Sources

The frequency of updates, accuracy and types of data, and data presentation were all considered prior to selecting each publicly available data source. After consideration of these criteria, six data sources were selected: COVID-19 Canada Open Data Working Group, Johns Hopkins University, Statistics Canada, Government of Canada, COVID Canada Spreadsheet, and Alberta Health COVID-19 Alberta Statistics [4-9].

The data from COVID-19 Canada Open Data Working Group contains detailed information on each confirmed case in Canada [4, 10]. Each case is listed with the reported date, province, and health region within the province. Additional demographic information such as age and sex are provided for some cases. Travel history and method of viral transmission are also included in some instances. This data shows new cases of COVID-19 in each province/region per day, in addition to the total cumulative cases.

Time-series data for many countries/regions are used from Johns Hopkins University [5]. The one time-series is for confirmed cases, and another is for fatalities caused by the virus. Members of our team have gathered policy data for many countries/regions. By combining the policy data with the time-series data, visualizations of how policies may influence the spread of the virus were created.

Statistics Canada provides data on the severity of cases [6]. This allows for the determination of the percent of cases that end up as hospitalizations and in the intensive care unit, as well as the percent of cases that result in fatalities.

Data from the government of Canada includes the number of daily cases, tests for COVID-19, deaths, and recoveries for each province [7]. This allows for visualizations of how prevalent testing is across the provinces and helps to make sense of surges of case numbers.

The COVID Canada spreadsheet provides varying information for each of the provinces, as well as data for all of Canada and other parts of the world [8]. Common data includes number of cases, tests, recoveries, hospitalizations, and deaths. This data is tabulated on the Tracker to provide an easily accessible means to see all of the raw data in one place. This data is also used to create a cumulative sum figure of cases/hospitalizations/ICU admissions past a standard deviation from the mean.

The COVID-19 Alberta Statistics from Alberta Health provides detailed information on cases, severe outcomes, and testing efforts within Alberta [9]. These data have been used to generate the Alberta tab of the COVID Tracker. This tab was created in order to provide the requested information for policy makers within Alberta.

The data on interventions that were imposed to try to limit the spread of COVID-19 in Canada and other countries were collected from daily scans of news articles, websites, and the scientific literature were sorted into a spreadsheet. Information from accredited news agencies on international interventions were sorted into one of nine categories: changes to governance/law, financial interventions (debt relief, aid packages), service closures, travel/transit suppression (border closures), screening and testing, information dissemination, equipment/supplies/resource mobilization (hand sanitizer, mask manufacturing), quarantine/isolation, and wellness interventions (handwashing, masking). Official national, regional, and (in the case of localized outbreaks) municipal websites were searched and cited for measures put in place to stop the spread of and limit the exposure to COVID-19. MEDLINE searches using the terms “COVID-19, coronavirus, intervention, measures” and related keywords were limited to the year 2020. The date the intervention was put into effect was used to overlay the information on the Tracker site. These data sources were used to create various visualizations and analyses in the Tracker, which poses a challenge. To streamline the daily data update process, a set of algorithms was used to automate the data cleaning and data processing for each source. These data pre-processing algorithms have been put into use and improved over time to ensure they are resilient enough to handle data quality variances from the differing data sources.

### Missing Variables

There are missing values, or values that contradict previous values within each data source (e.g. total positive case numbers going down from one day to the next). These errors may be fixed by the data source. The data sources are pulled daily in order to obtain any corrections that are made. For time-series data with missing entries, the last observation carried forward method was used. Some datasets have different levels of granularity for different provinces. For example, the total number of ICU admissions is available for Alberta, but not the other provinces. As a result, figures that rely on this data do not have the option to switch provinces. As more data becomes available, the site will continually be updated in order to provide as much information as possible.

### Website Architecture

The Tracker website was created and hosted online to allow for the use of both stakeholders and the general public. The public facing end of the Tracker was created using the React framework for JavaScript. The data is received via RESTful APIs from a backend server built using the Django framework for Python. The purpose of the backend server is to aggregate all of the data and send only the necessary information to the client. Some of the data sources list each case individually, and as the number of cases grows, so does the size of the data files. Sending these to the browser for processing when a user visits the dashboard would create a delay in the figures appearing.

## Results

The final Tracker can be found at this website: https://www.chi-csm.ca/. The website offers six distinct tabs or pages that display 1) Alberta data, displayed in graphics (as described below), 2) Canadian and global data, displayed in graphics (as described below), 3) the modelling approach, 4) a tab that describes the data used, 5) the team members who were involved in the development of the Tracker, and 6) an “About” tab that describes COVID-19, the objectives of the Tracker and nuances of the data used. The data used for the Tracker is updated daily based on publicly available data sources. The Tracker currently has had over 25,000 unique visits (August 19, 2020), with many repeated visits from key stakeholders.

**Figure 1: Confirmed Cases of COVID-19 in the Selected Province:**
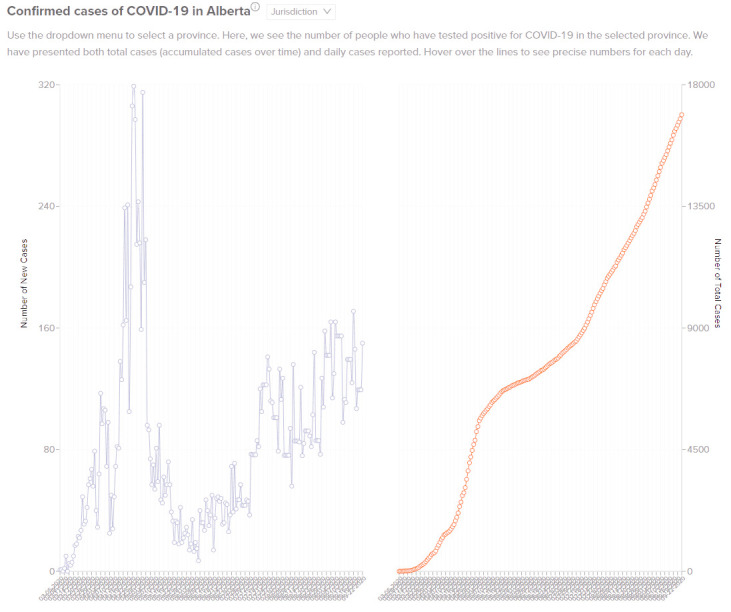


In Figure 1, the number of daily new cases for a selected provincial jurisdiction is charted on the left along with the cumulative number of total confirmed cases on the right. This graph helps to inform the overall trend of new COVID-19 cases in a provincial jurisdiction. For example, Canada’s initial peak of new cases occurred on April 23rd with 319 new cases in one day. With the addition of the Alberta tab, a similar figure is available where the viewer can select to see cases by health zone within Alberta.

**Figure 2: Cases by Health Region in the Selected Province:**
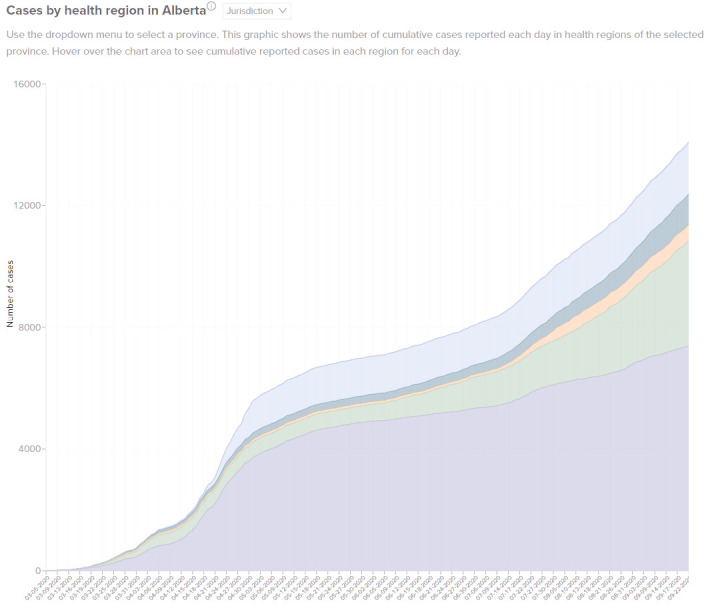


Figure 2 stacks the total case counts by health region in a Canadian province, which helps to highlight the differences in the COVID-19 trend between various health regions. Of note, each health region had a different experience, highlighting the disparate spread of COVID-19 within one province.

**Figure 3: International Policy Comparison:**
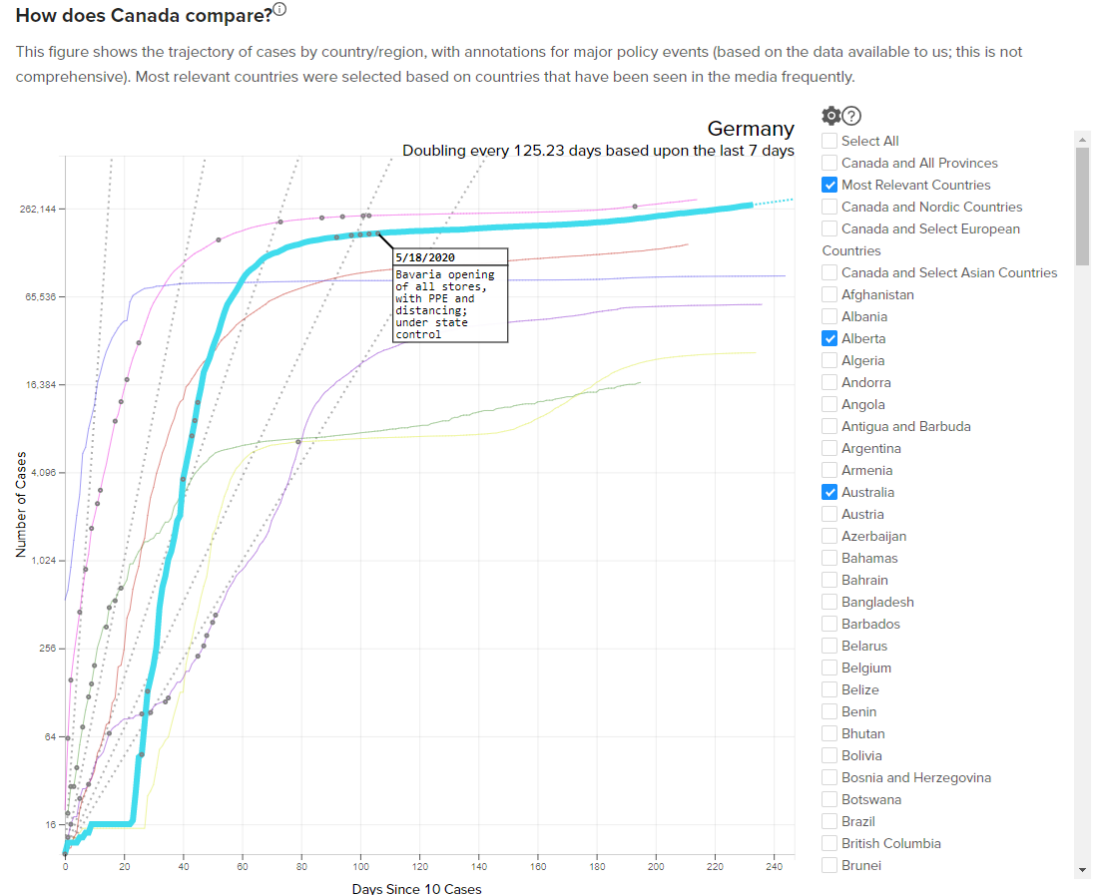


Comparisons of various international jurisdictions can be seen in Figure 3. For a few countries deemed to be relevant comparisons for Canada, annotated policy changes (e.g. border closures, bans on gatherings of groups of people) are available and are indicated on the figure. This custom-built figure enables decision-makers to see what public health interventions have been applied around the world, when those actions were taken relative to the arrival of COVID in that country. The graphic also allows the viewer to visualize how other governments’ COVID-19 policies have impacted the case trend. It should be noted that out of the elements of our search strategy, and due to the slow cycle of publishing in the academic literature, all of the interventions reported on the Tracker were sourced from news sites or government websites, and not academic publications.

**Figure 4: Severe Cases of COVID-19 in Canada:**
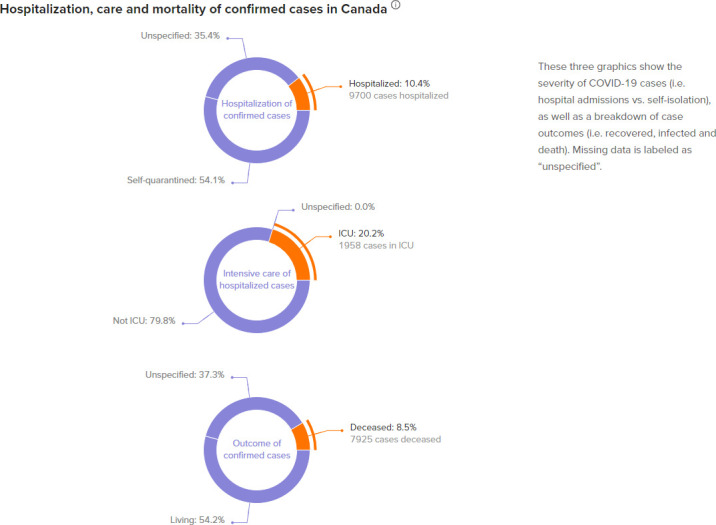


The overall hospitalization, ICU, and mortality proportions of confirmed COVID-19 cases in Canada can be observed in Figure 4. This graph paints a picture how COVID-19 may impact our healthcare system.

## Discussion

Building a user-friendly yet informative dashboard can be a daunting task and requires a diverse team of experts to collaborate. The University of Calgary’s COVID-19 Task Force was assembled and included biostatisticians, epidemiologists, software engineers, health economists, infectious disease experts, and health services researchers to successfully build a COVID-19 Tracker. This Tracker has been a useful tool in both keeping the public informed and guiding the decisions of policy-makers in Alberta and Canada. The information displayed in the Tracker enables key stakeholders to make decisions and policy changes using real-time data that protect the health of Albertans and more broadly, Canadians. The data were quickly collected from publicly available sources in order to develop advanced algorithms and user-friendly graphics.

### Other Dashboards

The CHI COVID-19 Tracker is one of the many dashboards that were designed in response to the quick and wide spread of the coronavirus [11]. One of the trailblazing teams was at the Center for Systems Science and Engineering at Johns Hopkins University, who launched their dashboard less than one month after the disease was first reported in Wuhan [12, 13]. In addition, the World Health Organization, and even well-known software developing companies (SAS Institute and Tableau), developed their own web-based trackers to monitor the ongoing pandemic at a global scale [14-16]. At a national level, country-wide surveillance is carried out by national Public Health Agencies and Ministries of Health, as well as major universities, like in Canada [17-20]. And at a more granular level, dashboards focused on specific Canadian provinces have been developed too [21, 22].

Most of these dashboards are updated on a daily basis and have interactive graphs with a hover function to reveal more information, like the CHI COVID-19 Tracker. Of note, the interactive features of our Tracker allow users to customize the figures by jurisdiction and the type of data shown (cases, hospitalizations, ICU admissions). Consequently, the geographical scope of our Tracker has improved granularity, as it displays data at the national and provincial level, and even by health regions. Another unique and novel characteristic of our Tracker is that it displays annotations for major policy changes that were implemented to slow the spread of COVID-19. This overlay of the policies on the country-specific curves helps inform policy- and decision-makers on how these initiatives may impact the case trend/COVID trajectory. 

### Strengths & Lessons Learned

The rapid unfolding of the COVID-19 situation calls for constantly evolving surveillance needs. It was essential to meet these demands quickly in order to allow for decisions to be made based on the most relevant data. Many figures were created at the start of the pandemic, and as we went through the peak and re-opening phases, new figures became necessary. To fulfil the evolving needs of the public, we have enhanced them over time through iterative design.

However, our Tracker is not without its limitations. Many of these come from the data sources, which have varying levels of quality and types of data available. For example, a figure on the number of active cases is helpful for policy-makers. This was originally not available on the Tracker, as this information was not included in any of the data sources available to us. The active cases could be computed from the number of total cases, fatalities and recoveries; however, total cases and fatality data was available at the provincial level, while recovery data was only provided for Canada as a whole. Thus, differences in the granularity of the data sources prevents us from presenting certain information. However, with the addition of another data source, a figure displaying the active cases in Alberta was created. Similarly, the number of cases detected is dependent on the number of individuals tested. As such, the data that is reported within the Tracker may over-represent those populations that are at a greater risk of disease compared to the general population. A full description of the limitations of the data is presented on the Tracker website, under the “About” tab. Ultimately, the quality of the data collected and cooperation between organizations that gather and report data play an essential role in the quality of the data reported. Accordingly, as we have been working with those that produce the data in Alberta, we have given feedback directly on the data throughout the pandemic: by visualizing and using it, we have been uncovering errors and helping them to find ways to improve it.

We want to emphasize the importance of establishing data formats early on in the data analysis process and prepare for the fact that data granularity changes over time. This would reduce the need to make changes and modifications later on. Further, standardizing data variables from different sources would improve quality and accuracy of analysis (e.g. consistent age categories). One indirect benefit of developing the Tracker was that errors in data were identified and relayed to the organizations responsible, inadvertently improving on the quality of data used.

### Knowledge Translation

To date, our work at CHI on the COVID-19 Tracker has been featured on the front page of the Calgary Herald and in the Edmonton Sun, and on radio shows including CBC Calgary Eyeopener, 770 CHQR, and CBC Edmonton; in online forums with over 1000 attendees; and it has been demonstrated to the Mayor of Calgary and his senior staff on multiple occasions (approximately 15 meetings). It has also been shared with the head of Alberta Health Services, Alberta Health, and the Deputy Head of Health Canada, many other policy-makers across Canada, and even the National Institutes of Health in the United States. Finally, our COVID Tracker provides real-time capture of COVID-19 testing to Albertans. By displaying models within our Tracker, we are poised to accurately evaluate the impacts of interventions and forecast trends. We are able to provide meaningful and timely information to policy-makers and the public on the COVID-19 pandemic situation, to keep our province informed and educated. The Tracker has been used to inform policy decisions in the City of Calgary’s response, including closing certain businesses early on in the pandemic. The Tracker has also supported the decision-making process about relaxing restrictions as the case numbers stabilized, such as allowing the reopening of businesses, and more recently the decision to mandate mask use in all public spaces in the city. All in all, the Tracker has been proven to be valuable and trustworthy to decision makers, and will thus continue to play a major role as other vital decisions are made (e.g., the decision to re-open public schools).

## Conclusion

It is essential that high quality data is used for evidence-informed decisions to ultimately minimize the economic consequences and lives lost from the COVID-19 pandemic. The COVID-19 Tracker offers digestible data that we will continue to share with Canada and other countries around the globe. Further, the Tracker superimposes policy decisions on case curves to add depth and possible interpretation of data trends. We expect the Tracker to continue evolving and improving, in order to answer questions that result from the ongoing uncertainty the pandemic brings. For example, many countries around the world began to reduce or remove interventions designed to stop the spread of the virus, in an attempt to return to pre-pandemic conditions. With the goal of avoiding a “second wave”, it is important to look at how these relaxations affect the countries that implement them. This work has the potential to shape policies nationally and internationally, and we aim to provide the much-needed roadmap for global efforts to alleviate the COVID-19 burden.

## Acknowledgments

We would like to thank all team members at CHI who worked on the Tracker for their contributions and valuable insights in the development, updates and revisions of the website and Tracker.

## Author contributions

AK and OC managed, analysed, and maintained the data, engineered the website, created the graphics, and participated in the writing and editing of the manuscript. LOV and CD wrote and edited website captions and content, and assisted with the drafting and editing of the manuscript. VA contributed to the writing and editing of the manuscript. SK guided the visual design of the website. CE and TW guided the construction of the website. DS, NS and TW participated in the University of Calgary’s COVID-19 Task Force and development of the features of the Tracker. All authors reviewed and approved of the manuscript.

## Ethics statement

Ethical approval was not required, as the study relied on publicly available data.

## Abbreviations

CHI	Centre for Health Informatics
COVID-19 or COVID	Coronavirus Disease
SARS-CoV-2	Severe Acute Respiratory Syndrome CoronaVirus 2
